# Smart Paper-Based Nanosensor for Simultaneous Environmental eMonitoring of Nitrate and Nitrite

**DOI:** 10.1021/acsmeasuresciau.5c00122

**Published:** 2025-10-30

**Authors:** Mahdi Oroujlo, Zeinab Bagheri, Tina Naghdi, Hamed Golmohammadi

**Affiliations:** † Department of Cell & Molecular Biology, Faculty of Life Sciences and Biotechnology, 48512 Shahid Beheshti University, 1983969411 Tehran, Iran; ‡ Nanosensor Bioplatforms Laboratory, 113401 Chemistry and Chemical Engineering Research Center of Iran, 14335-186 Tehran, Iran

**Keywords:** Nitrate and nitrite detection, on-site monitoring, paper-based sensors, smart sensors, carbon dots, point-of-need

## Abstract

Nitrate (NO_3_^–^) and nitrite (NO_2_^–^) contamination, primarily from agricultural fertilizers, food additives, and industrial effluents, poses a significant threat to the environment and public health. While current detection methods are sensitive, they often rely on complex instrumentation, trained personnel, and time-consuming procedures, limiting their applicability in field settings. To overcome these limitations, we developed a portable, IoT-integrated, paper-based fluorescent sensor that meets all WHO’s REASSURED criteria for ideal sensing devices. The sensor employs phenylene-diamine-derived carbon quantum dots (CQDs) as fluorescent probes with NO_2_^–^ inducing fluorescence quenching via nitrosylation and diazotization. For simultaneous detection, NO_3_^–^ is converted in situ to NO_2_^–^ through a simple, eco-friendly on-strip reduction step, enabling unified quantification of both analytes. The platform achieved a limit of detection (LOD) of 0.1 ppm and exhibited a linear response from 1 to 100 ppm. Its performance was validated by using real water samples, successfully determining both NO_3_^–^ and NO_2_^–^ concentrations. Integrated with a custom hand-held optoelectronic reader and smartphone interface, the system enables real-time data acquisition, wireless transmission, and rapid on-site decision-making. This green, low-cost, and efficient platform offers a practical solution for environmental eMonitoring by integrating nanotechnology, paper based analytical device and smart sensing in a single device.

## Graphical abstract

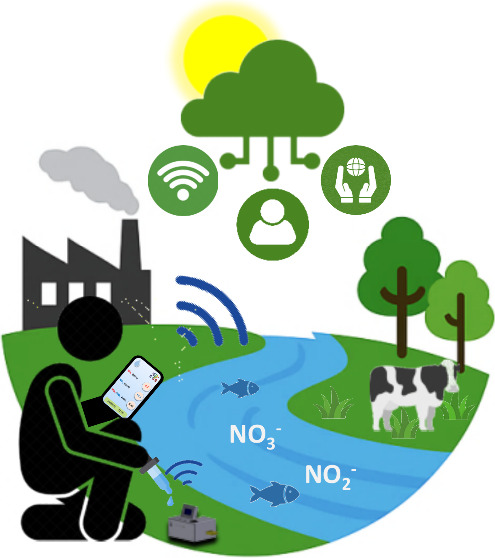

## 1 Introduction

Human activities, especially extensive use on nitrogen fertilizers, food additives, and industrial waste, are the main reason nitrate (NO_3_^–^) and nitrite (NO_2_^–^) are now major inorganic pollutants found extensively in the environment and raising public health concerns. Although nitrates play a vital role in plant nutrition, their excessive accumulation in ecosystems, and especially their conversion into nitrites under certain conditions (e.g., low pH, the presence of reducing bacteria, or elevated temperatures), poses serious health risks. NO_2_^–^ is significantly more toxic and reactive than NO_3_^–^ and is associated with the formation of carcinogenic nitrosamines, methemoglobinemia (particularly in infants), and various blood and neurological disorders. in this regard, Maximum allowable concentrations (MAC) for NO_3_^–^ (50 ppm) and NO_2_^–^ (3 ppm) in drinking water have been established by the WHO and EU to mitigate these risks.^1,2^

Given the critical importance of NO_3_^–^ and NO_2_^–^ detection, it is essential to develop rapid detection methods alongside the high-precision analytical systems used in regulatory laboratories. Current techniques—such as spectrophotometry, chromatography, and electrochemistry—offer high sensitivity and accuracy but are often time-consuming, depend on sophisticated equipment, and necessitate trained personnel.^3,4^

To address these limitations, the development of rapid sensors for the detection of NO_3_^–^ and NO_2_^–^ has become a pressing priority. These sensors must go beyond simple detection; they should meet the REASSURED criteria: Real-time connectivity, Ease of specimen collection, Affordability, Sensitivity, Specificity, User-friendliness, Rapidness and robustness, Equipment-free operation (or minimal), and Deliverability to end-users.^5–7^

A promising strategy for rapid and accurate detection involves integrating fluorescent readers with Paper-Based Analytical Devices (PADs). Paper, composed of hydrophilic cellulose fibers, is a low-cost, lightweight, flexible, tailorable, porous, and versatile biopolymer. Recently, PADs have attracted significant interest for point-of-care diagnostics and environmental monitoring applications due to their affordability, ease of fabrication and use, and overall efficiency. This growing attention stems from paper’s many advantageous properties over traditional sensor substrates, including its widespread availability, cost-effectiveness, nontoxicity, biodegradability, sustainability, foldability, and excellent capillary properties.^5,8–10^ The combination of PADs with fluorescent readers merges the affordability and portability of PADs with the high sensitivity and selectivity of fluorescence-based detection. The system is designed for both qualitative and quantitative analyses, with the reader easily tailored to detect specific analytes by incorporating different recognition elements. One of its major strengths is suitability for point-of-care (POC) use. The reader can connect to a smartphone, allowing real-time data collection, on-site interpretation, and instant result sharing.^5,11–13^

In particular, the simultaneous detection of NO_3_^–^ and NO_2_^–^ is of strategic importance. The dynamic interconversion between these two ions—affected by microbial activity, temperature, pH, and oxygen levels—not only influences toxicity but also serves as a diagnostic tool for identifying the source and type of pollution.^14^ For instance, a higher NO_2_^–^-to-NO_3_^–^ ratio may suggest recent microbial activity or contamination from untreated sewage or industrial runoff, while elevated NO_3_^–^ levels without NO_2_^–^ may indicate fertilizer overuse or agricultural leaching. Therefore, a sensor capable of real-time, simultaneous detection of both compounds could provide critical insights into environmental conditions, allowing for more responsive interventions and targeted remediation strategies.^15^

Herein, we developed a portable fluorescent sensor for simultaneous optical monitoring of NO_3_^–^ and NO_2_^–^ in water samples, which consists of a fluorescent sensing probe (carbon quantum dots (CQDs)) immobilized within a paper substrate.

CQDs are a type of zero-dimensional carbon-based nanomaterial recognized as one of the most promising and widely studied luminescent nanoparticles in optical sensing applications. Their broad use in (bio)­sensing platforms stems from their outstanding physicochemical and photostability, stable and tunable fluorescence properties, cost-effectiveness, ease of synthesis and surface modification, natural abundance, excellent water dispersibility, significantly lower toxicity compared to conventional QDs, and strong resistance to photobleaching.^16,17^

In this study, we first synthesized and characterized phenylene-diamine-derived CQDs. These CQDs exhibit strong fluorescence and possess aromatic amine functional groups, which are crucial for their selective interaction with NO_2_^–^.^18,19^ Under mildly acidic conditions, NO_2_^–^ reacts with these groups via nitrosylation and diazotization, producing diazonium and N-nitroso compounds that effectively quench the fluorescence of the CQDs.^18,20^

To enable simultaneous detection of NO_3_^–^ and NO_2_^–^, we incorporated a simple, eco-friendly chemical reduction step that converts NO_3_^–^ into NO_2_^–^ directly on the sensing substrate. This allows both analytes to be detected through the same fluorescence quenching mechanism, streamlining the sensing process.

For practical applications, the CQDs were integrated into a PAD in a strip format. These strips were placed in a custom-designed holder compatible with the detection system. To quantify the fluorescence signals, we fabricated a portable, hand-held optoelectronic reader equipped with Internet of Things (IoT) capabilities. This reader enables real-time fluorescence measurement, wireless data transmission, and user-friendly operation, facilitating rapid, on-site NO_3_^–^ and NO_2_^–^ monitoring.

By combining nanomaterial-based sensing, green chemistry, and IoT-enabled hardware, this platform provides a low-cost, portable, efficient, and smart solution for the real-time environmental analysis of NO_3_^–^ and NO_2_^–^ contamination.

## 2 Experimental Section

### 2.1 Reagents and Equipment

Sodium nitrate (NaNO_3_), sodium nitrite (NaNO_2_), zinc (Zn), o-phenylenediamine (o-PDA), sodium hydroxide (NaOH), hydrochloric acid (HCl) 37%, calcium sulfate (CaSO_4_), mercury (ll) chloride (HgCl_2_), manganese­(ll) sulfate (MnSO_4_), copper (ll) chloride­(CuCl_2_), magnesium chloride­(MgCl_2_), lead­(ll) acetate (Pb­(C_2_H_3_O_2_)_2_), nickel­(ll) acetate­(C_4_H_6_NiO_4_), and cadmium chloride (CdCl_2_) were purchased from Merck. Milli-Q grade water (18.2 MΩ resistance) was used in all experiments. Filter paper (Whatman no. 1) was purchased from Whatman International, Ltd. (England). A waterproof sticker was purchased from a local market in Iran.

The fluorescence measurements were executed by a Cary Eclipse (Agilent) spectro-fluorometer. TEM images of the fabricated CQDs were performed by a transmission electron microscope (Zeiss-EM900). FT-IR spectrum was recorded on an FT-IR spectrometer (Thermo Nicolet). The 3D printing process was carried out with a prusa mark3 slicer.

### 2.2 Fabrication of Paper-Based Nanosensor

The developed paper-based nanosensor, as shown in Figures 1A and 3A, consists of two main parts: two test zones (CQDs immobilized on paper substrates) and two paper channels (a Zn-free paper channel connected to the NO_2_^–^ test zone and a Zn-embedded paper channel (reduction zone) connected to the NO_3_^–^ test zone), all of which are attached to the adhesive side of a waterproof sticker.

**Figure 1:**
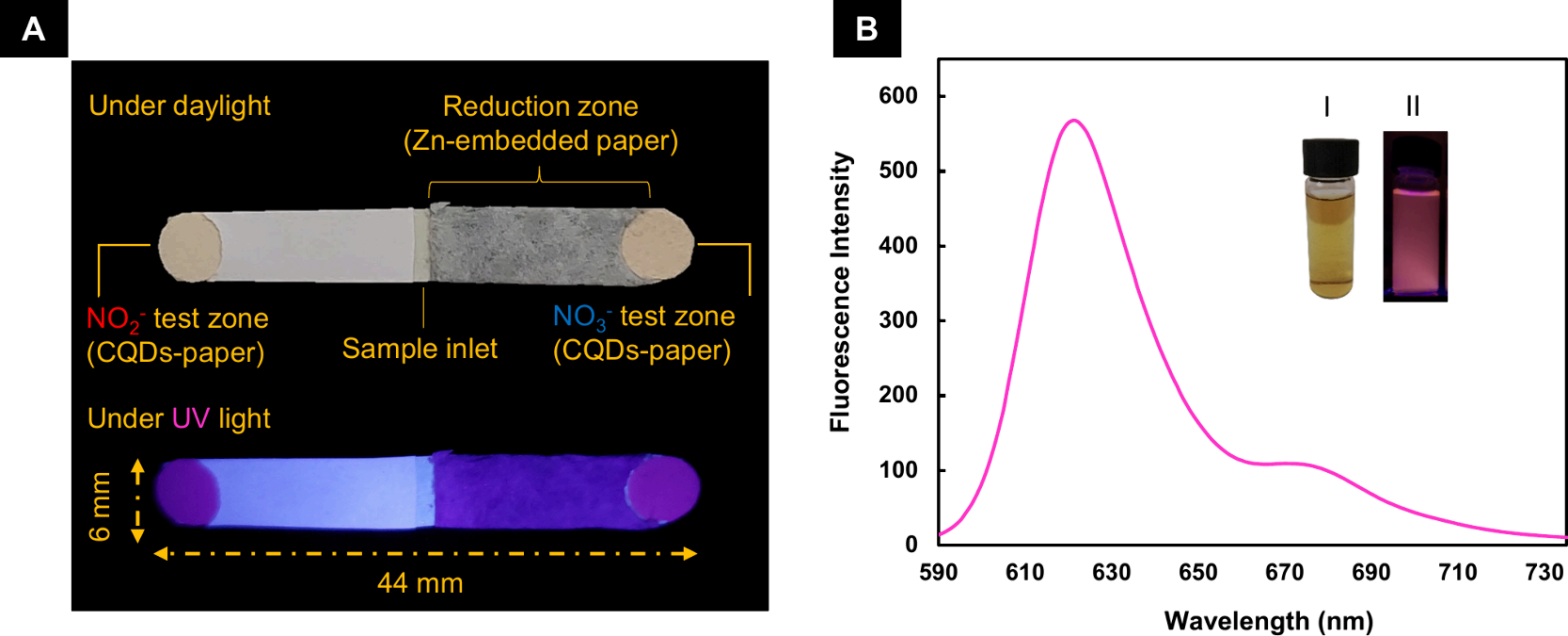
(A) Images of the fabricated paper-based nanosensor with its test zones and reduction zone under daylight and UV light. (B) Fluorescence spectrum of the synthesized CQDs. The insets are the optical images of the fabricated CQDs illuminated under (I) daylight and (II) UV light.

#### 2.2.1 Synthesis of Fluorescent Carbon Quantum Dots

Fluorescent CQDs were synthesized via a hydrothermal method. 0.15 g of o-PDA as a carbon source was dissolved in 10 mL of water and then placed in an ultrasonic bath for 6 min. The reaction mixture was then transferred to a 25 mL autoclave (Teflon-lined stainless-steel) and heated in an oven at 195 °C for 12 h. Following that, the synthesized CQDs were centrifuged for 15 min at 4500 rpm. The supernatant was then separated and stored in the dark at 4 °C before use. The fluorescence spectrum and optical images of the synthesized CQDs (under daylight and UV light) are presented in Figure 3B. The TEM image, size analysis, and FT-IR spectra of the synthesized CQDs are also presented in Figures S1–S3, respectively.

#### 2.2.2 Fabrication of the Paper Nanosensor Test Zones

The test zones of the paper-based nanosensor for optical sensing of NO_3_^–^ and NO_2_^–^ (Figure 1A) were fabricated as follows: 50 pieces of the filter paper (Whatman no. 1) previously punched using a hole punch into circles of 6 mm diameter were immersed in 5 mL of the synthesized CQDs solution (diluted with water in a 1:3 ratio) for 5 min. The paper pieces were then separated from the solution and air-dried at room temperature. To reduce the coffee ring effect and increase the sensor efficiency and reproducibility, the paper pieces containing embedded CQDs were rinsed again with water, air-dried, and finally stored in a dry and dark place before use.

#### 2.2.3 Fabrication of the Paper Nanosensor Reduction Zone

The reduction zone of the paper-based nanosensor for the reduction of NO_3_^–^ to NO_2_^–^ (Figure 1A) was fabricated as follows: A filter paper (20 cm × 20 cm) was immersed in a suspension of zinc metal powder (60 mg) in water (10 mL) for 2 h to prepare a zinc-containing paper pulp. After immobilization of zinc metal in the paper tissue, which was confirmed by the paper turning gray, the zinc-embedded paper pulp was cast onto a glass and then air-dried at room temperature, and finally cut into pieces of the desired size and stored in a dry place before use.

### 2.3 Design and Fabrication of Smart Hand-Held Optical Analyzer

The housing of the smart analyzer was designed by using SolidWorks 2020 and fabricated via 3D printing. The design includes designated compartments for key components: the AS7341 spectral sensor (3.3–5 V, 23 mm × 30.5 mm, Adafruit, China), an Arduino ESP32 IoT-enabled microcontroller (3.3 V, 28 mm × 55.3 mm, China), a surface-mounted UV-LED (2835 model, 3–3.6 V, 0.1 W, China), an Arduino organic light emitting diode (OLED) display module (bicolor, 0.96″, 128 × 64 pixels, China), a lithium-polymer (LiPo) rechargeable battery (18650 model, 2600 mAh, 3.7 V, 18 mm × 65 mm, China), a micro-USB charging module (KC 864–2A, 5 V, 5 W, China), and a 3D-printed sample holder (15 × 55 × 3 mm), which well-embedded for the paper sensor. Each component was precisely mounted in its allocated position within the dark chamber to optimize the optical performance and minimize ambient interference. System functionality and communication protocols were programmed using an open-source Arduino software (Integrated Development Environment (IDE)).^5,11–13,21^

### 2.4 Recommended Procedure for Optical Determination of NO_3_^–^ and NO_2_^–^ Using the Developed Sensor

The optical determination of NO_2_^–^ and NO_3_^–^ was carried out using the developed sensor according to the following experimental procedure. For NO_2_^–^ determination, 2 mL samples of each NO_2_^–^ concentration in the range 0–100 ppm containing 100 μL of 100 mM HCl were first prepared. The paper nanosensor was then inserted in its holder. For each NO_2_^–^ concentration, 10 μL of the relevant prepared solution was dropped onto the sample inlet (Figure 2A) of the paper sensor holder. After 30 min, the paper sensor holder was placed inside the fabricated smart analyzer in order to record the fluorescence and color intensity of the NO_2_^–^ test zone in the 670 nm optical channel of the analyzer. Using the analyzer’s IoT module, the recorded optical signals were finally transmitted wirelessly to a smartphone to quantify the corresponding NO_2_^–^ concentrations via the associated determination algorithm/equation using our self-developed mobile’s app (Figure 2D). It is noteworthy that the system’s sensing strategy for NO_3_^–^ monitoring relies on the in situ reduction of NO_3_^–^ to NO_2_^–^ by zinc; thus, the NO_3_^–^ concentration is indirectly quantified based on the detected NO_2_^–^ signal in the NO_3_^–^ test zone following the passage of the NO_3_^–^ solution through the reduction zone (Zn-embedded paper channel). Accordingly, for NO_3_^–^ determination, the above procedure was performed on samples of each NO_3_^–^ concentration in the range of 0–100 ppm containing 100 μL of 100 mM HCl, after which the fluorescence intensity/color of the NO_3_^–^ test zone was read in the 670 nm optical channel of the analyzer and finally quantified using the associated determination algorithm/equation.

**Figure 2:**
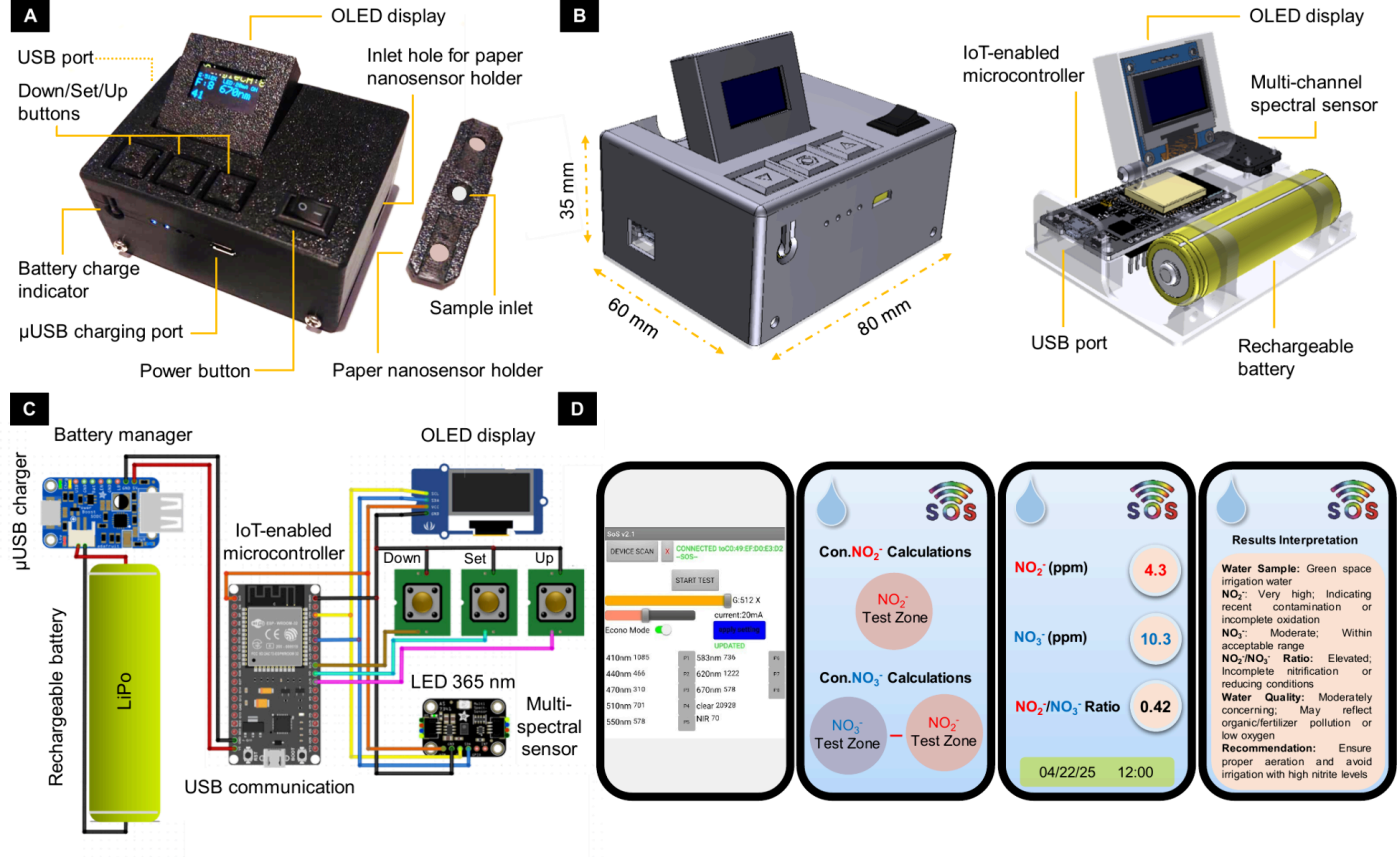
(A–C) Images of the fabricated smart hand-held optical analyzer, its components, and its schematic electronic diagram. (D) Schematic images of the self-developed mobile’s app: from left to right, the spectral data of the fabricated smart hand-held optical analyzer in each optical channel, the steps of spectral data formulation in the optical channel of 670 nm to quantify the NO_2_^–^ and NO_3_^–^ concentrations using the related equations, and the interpretation of the results obtained for NO_2_^–^, NO_3_^–^, and their ratio.

### 2.5 Real Samples Analysis

The practical applicability of the developed sensor in real sample analysis was evaluated by simultaneous monitoring of NO_2_^–^ and NO_3_^–^ in different water samples. Eight different water samples (tap water, groundwater, river water, aqueduct water, mineral water, lake water, green space irrigation water, and sterilized water (for formula milk) samples), without any pretreatment or sample preparation process, were subjected to the recommended procedure for optical determination of NO_2_^–^ and NO_3_^–^ by using the developed sensor. Each of the real water samples was also separately spiked with concentration levels of 10 ppm of NO_2_^–^ and NO_3_^–^, and then analyzed according to the recommended procedure in Section 2.4. It is noteworthy that, given the system’s sensing strategy and the presence of both NO_3_^–^ and NO_2_^–^ in the water samples examined, the corresponding NO_3_^–^ concentration for each real water sample was calculated by subtracting the measured NO_2_^–^ concentration in the NO_3_^–^ and NO_2_^–^ test zones, respectively.

## 3 Results and Discussion

The smart optical sensor system for simultaneous monitoring of NO_3_^–^ and NO_2_^–^ includes a paper-based nanosensor (CQDs immobilized within a paper substrate), a smart hand-held optical analyzer, and a self-developed mobile’s app.

### 3.1 Optimization of Effective Variables on the Efficiency of the Fabricated Paper-based Nanosensor

The main variables influencing the performance of the fabricated paper-based nanosensor, including the pH, the amount of zinc immobilized on the paper substrate, and the reaction time, were examined to enhance its efficiency and determine the optimal operating conditions. As illustrated in Figure S4, the fluorescence intensity of the CQDs immobilized on the paper substrate of the fabricated sensor is decreased with lowering pH to 4 in the presence of 20 ppm of NO_2_^–^, reaching maximum quenching within the pH range of 2–4. This observation supports the proposed mechanism for the fluorescence quenching of CQDs by NO_2_^–^ in an acidic medium (Section 3.3). Under these acidic conditions, NO_3_^–^ is also efficiently reduced to NO_2_^–^ by zinc; therefore, pH = 3 was chosen as the optimal pH in the procedure.

The effect of zinc loading in the sensor’s reduction zone on NO_3_^–^-to-NO_2_^–^ conversion and sensor performance was also investigated. As shown in Figure S5, complete NO_3_^–^ reduction was achieved with a 6 g/L zinc suspension; thus, paper channels were immersed in this suspension during fabrication of the reduction zone (Section 2.2.3).

During this investigation, we observed that the response of our developed sensor is time-dependent. As shown in Figure S6, the fluorescence intensity of CQDs immobilized on the paper substrate is decreased over time in the presence of 20 ppm of NO_2_^–^, eventually reaching a stable value after 30 min. Therefore, an equilibrium time of 30 min was selected as the optimal duration in the recommended procedure for NO_3_^–^ and NO_2_^–^ determination using the developed sensor (Section 2.4).

### 3.2 Smart Hand-Held Optical Analyzer

The core component of the fabricated smart hand-held optical analyzer is the Adafruit AS7341, a compact, high-performance multispectral sensor that analyzes and detects light across a broad spectral range. This 11-channel spectrometer comprises 8 channels covering the visible spectrum (400–700 nm), alongside 3 auxiliary channels (clear, near-infrared, and flicker). Integrated with 6 parallel analog-to-digital converters (ADCs), it enables efficient and accurate signal acquisition. The sensor features high accuracy and sensitivity in light analysis, compact dimensions, and low power requirements, making it ideal for portable analytical platforms. Communication with the sensor is established using the Inter-Integrated Circuit (I^2^C) protocol, which simplifies wiring by requiring only two bidirectional lines and enables the control of multiple peripheral devices from a single master controller. This efficient communication architecture supports flexible data rates and compatibility with various microcontrollers. Thanks to these capabilities, this multispectral sensor has found various applications in areas such as color-based shopping/search, smart building ambient monitoring, light source calibration, laboratory analysis, as well as wearable and portable diagnostics devices.^5,11–13,21^

The fabricated analyzer incorporates an IoT-enabled microcontroller (ESP32), allowing wireless connectivity to IoT gateways through Wi-Fi or Bluetooth. This enables real-time transfer of analytical data to a smartphone for further processing via a dedicated mobile app, which applies in-app algorithms to quantify analytes concentration. Additionally, the fabricated analyzer is capable of performing onboard analysis and displaying the results directly on its built-in screen. These features support both standalone operation and seamless data sharing with remote devices for monitoring applications.

### 3.3 Optical Monitoring of NO_2_^–^ and NO_3_^–^

A “turn-off” fluorescence sensing strategy was used in the developed sensor for monitoring of NO_2_^–^ in water samples. As shown in Figure 3, the fluorescence of CQDs in solution (Figure 3C,D) and paper substrate (Figure 3B) is decreased/quenched by increasing the NO_2_^–^ concentration, which is primarily attributed to electron transfer between the CQDs and NO_2_^–^, particularly in acidic conditions. Indeed, under mildly acidic conditions, NO_2_^–^ can react with the electron-donating functional groups on the CQD surface through nitrosylation and diazotization, forming diazonium and N-nitroso compounds. These reactions facilitate electron transfer from CQDs to NO_2_^–^, resulting in fluorescence quenching. Protonation of NO_2_^–^ and formation of more reactive compounds in acidic environments, while facilitating this electron transfer, reduces the number of excited electrons in CQDs and consequently decreases their fluorescence.^18,20^ Therefore, in the recommended procedure for optical determination of NO_2_^–^ and NO_3_^–^ using the developed sensor (Section 2.4), hydrochloric acid (100 μL of 100 mM HCl) is added to water samples to ensure a suitable acidic environment for the sensing reactions.

**Figure 3:**
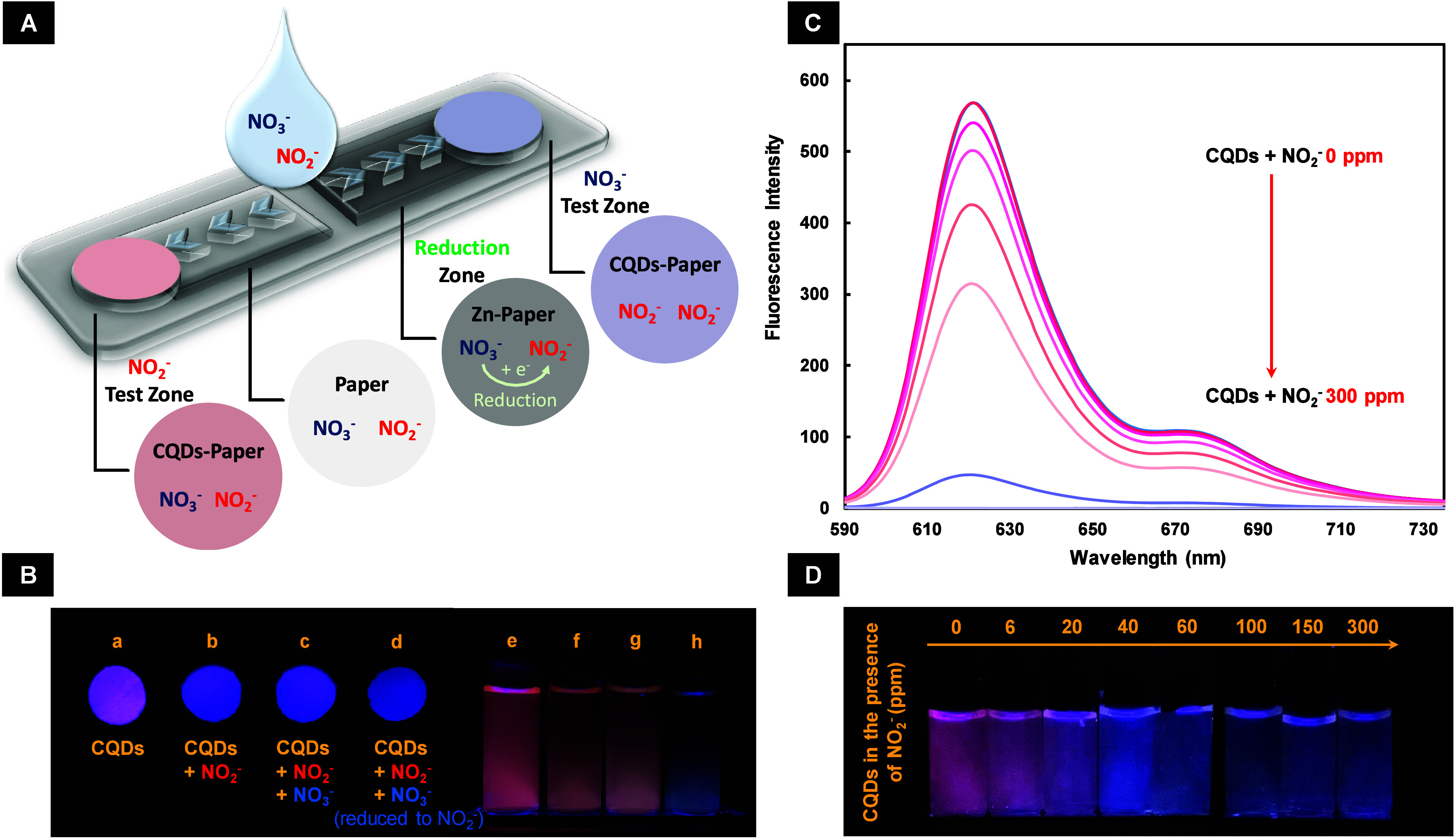
(A) Schematic image of the fabricated paper-based nanosensor, its components, and its sensing strategy. (B) Images of the synthesized CQDs in (a-d) paper substrate and (e-g) their corresponding images in the solution phase, in the absence of NO_2_^–^ and NO_3_^–^, in the presence of NO_2_^–^, in the presence of NO_2_^–^ and NO_3_^–^, and in the presence of NO_2_^–^ and NO_3_^–^ reduced after passing through the reduction zone (Zn-embedded paper) of the fabricated paper-based nanosensor, respectively from left to right. (C) Fluorescence spectra of the synthesized CQDs in solution with various concentrations of NO_2_^–^ in the range from 0 to 300 ppm, and (D) their corresponding images in glass vials.

On the other hand, as shown in [Fig fig3]Bc,g, the fluorescence of the synthesized CQDs remains unchanged in the presence of NO_3_^–^. Therefore, as schematically depicted in Figure 3A, for optical monitoring of NO_3_^–^, the developed sensor also incorporates a reduction zone consisting of a Zn-embedded paper channel through which the sample passes. In this zone, NO_3_^–^ is reduced to NO_2_^–^ by zinc under the acidic conditions described above, before reaching the NO_3_^–^ test zone (CQDs-paper), enabling indirect quantification of NO_3_^–^ alongside direct and simultaneous detection of NO_2_^–^ in water samples in two separate test zones.

### 3.4 Storage Time and Stability of the Developed Sensor

Considering the critical role of long-term stability in sensor reproducibility, performance, and practical applicability, the fluorescence of the fabricated paper-based nanosensor was monitored over time while being stored at room temperature in a dark, dry environment. As shown in Figure S7, the sensor’s fluorescence intensity remained essentially unchanged for up to 4 weeks, demonstrating its long-term stability and suitability for storage in practical applications.

### 3.5 Analytical Performance of the Developed Sensor

The developed sensor’s analytical characteristics for the optical determination of NO_2_^–^ and NO_3_^–^ were evaluated for sensitivity, linearity, and reproducibility. The calibration curve for the quantitative determination of NO_2_^–^ was plotted in the ranges of 1–100 ppm with a correlation coefficient (*r* ^2^) of 0.9716. The linear regression equation for NO_2_^–^ was *S = −1.4656C + 183.72*, where *S* is the color intensity and *C* is the concentration of NO_2_^–^(ppm) (Figure 4A). The relative standard deviation (RSD) for six replicate measurements of 25 ppm of NO_2_^–^ was calculated to be 2.4%, verifying the reproducibility of the developed sensor. The limit of detection (LOD) was experimentally determined as the lowest analyte concentration yielding a signal at least three times higher than the standard deviation of the blank (S/*N* ≥ 3). Based on replicate measurements (n = 6), the experimental LOD was found to be 0.1 ppm, which meets the MAC set by the WHO and EU for NO_2_^–^ (3 ppm) and NO_3_^–^ (50 ppm) in water samples.^1,2^ Our developed sensor results, as presented in Table 2, are comparable to those of other reported sensors for the determination of NO_2_^–^ and NO_3_^–^ in water samples.

**Figure 4:**
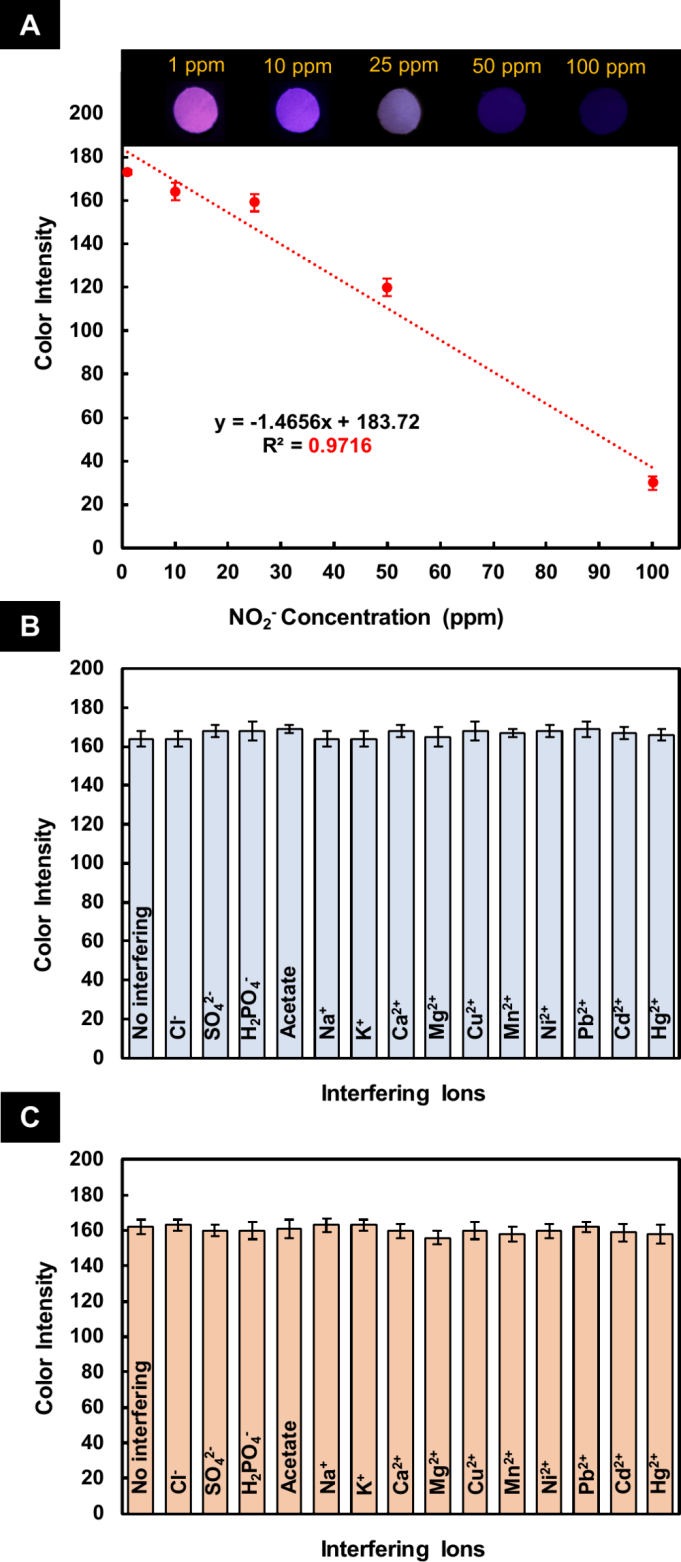
(A) Calibration curve for the fluorescence determination of NO_2_^–^ using the developed smart sensor, and (inset) images of its corresponding test zones. Independent evaluation of the developed sensor’s response for the determination of (B) 10 ppm of NO_2_^–^ and (C) 10 ppm of NO_3_^–^ in the presence of coexisting ions (Cl^–^ (1000 ppm), SO_4_^2–^ (1000 ppm), H_2_PO_4_^–^ (100 ppm), acetate (100 ppm), Na^+^ (1000 ppm), K^+^ (1000 ppm), Ca^2+^ (500 ppm), Mg^2+^ (500 ppm), Cu^2+^ (0.2 ppm), Mn^2+^ (0.2 ppm), Ni^2+^ (0.2 ppm), Pb^2+^ (0.2 ppm), Cd^2+^ (0.1 ppm), and Hg^2+^ (0.1 ppm)). No interfering: the developed sensor in the presence of NO_2_^–^/NO_3_^–^ without coexisting ions.

### 3.6 Interference Study

The selectivity of our developed sensor was independently evaluated for NO_2_^–^ and NO_3_^–^in the presence of some chemicals, probably existing in the water samples. The results of these investigations (Figure 4B and 4C), performed by competition experiments in the presence of typical interfering substances possibly present in water samples together with NO_2_^–^ and NO_3_^–^, illustrate that the presence of possible coexisting ions does not significantly affect (< ± 5%) the analytical signal of the developed sensor, validating its high selectivity toward both analytes (NO_2_^–^ and NO_3_^–^).

### 3.7 Application of the Developed Sensor in Real Samples Analysis

In order to validate the applicability of our developed sensor, its efficiency for the simultaneous monitoring of NO_2_^–^ and NO_3_^–^ in different water samples (tap water, groundwater, river water, aqueduct water, mineral water, lake water, green space irrigation water, and sterilized water (for formula milk) samples) was put into practice. The recovery values ranged from 91% to 121% (Table 1), confirming the satisfactory accuracy of the developed sensor. The results obtained for both unspiked and spiked water samples demonstrate that the developed sensor has strong potential for the on-site, simultaneous environmental monitoring of NO_3_^–^ and NO_2_^–^ at the point of need.

**Table 1:** Simultaneous Determination of NO_2_^–^ and NO_3_^–^ in Various Water Samples by the Developed Sensor

sample		analyte added (ppm)	analyte found^a^ (ppm)	recovery (%)
tap water (Tehran city, Punak area, Iran)	NO_2_^–^	-	3.4 ± 0.3	-
		10	13.7 ± 0.4	103
	NO_3_^–^	-	16.1 ± 0.6	-
		10	27.8 ± 0.5	117
tap water (Tehran city, Gisha area, Iran)	NO_2_^–^	-	5.0 ± 0.4	-
		10	15.2 ± 0.4	102
	NO_3_^–^	-	17.9 ± 0.8	-
		10	29.3 ± 0.5	114
tap water (Qazvin city, Iran)	NO_2_^–^	-	7.8 ± 0.6	-
		10	18.1 ± 0.8	103
	NO_3_^–^	-	20.5 ± 1.1	-
		10	32.2 ± 1.2	117
river water (Qazvin city, Iran)	NO_2_^–^	-	6.4 ± 0.2	-
		10	16.6 ± 0.3	102
	NO_3_^–^	-	17.6 ± 0.9	-
		10	29.3 ± 0.7	117
well water (Qazvin city, Iran)	NO_2_^–^	-	9.3 ± 1.0	-
		10	21.4 ± 1.3	121
	NO_3_^–^	-	16.1 ± 0.9	-
		10	27.8 ± 0.8	117
aqueduct water (Qazvin city, Iran)	NO_2_^–^	-	7.8 ± 1.1	-
		10	18.1 ± 1.0	103
	NO_3_^–^	-	17.6 ± 1.1	117
		10	29.3 ± 1.4	
mineral water (Miva Co.)	NO_2_^–^	-	N.D^b^	-
		10	9.1 ± 0.9	91
	NO_3_^–^	-	N.D	-
		10	10.3 ± 0.7	103
mineral water (Jajrood Co.)	NO_2_^–^	-	N.D	-
		10	10.8 ± 0.9	108
	NO_3_^–^	-	1.5 ± 0.3	-
		10	11.7 ± 0.5	102
lake water (Chitgar, Tehran city, Iran)	NO_2_^–^	-	4.9 ± 0.4	-
		10	16.6 ± 0.4	117
	NO_3_^–^	-	13.1 ± 0.9	-
		10	24.9 ± 1.1	118
green space irrigation water (Pardisan park, Tehran city, Iran)	NO_2_^–^	-	4.3 ± 0.7	-
		10	15.1 ± 0.6	108
	NO_3_^–^	-	10.3 ± 0.6	-
		10	21.9 ± 0.9	116
sterilized water for formula milk (Majan Co.)	NO_2_^–^	-	N.D	-
		10	9.3 ± 1.1	93
	NO_3_^–^	-	2.9 ± 0.8	-
		10	14.6 ± 0.5	117

a X ± ts/√n at 95% confidence (*n* = 3).

b ND, not detected.

## 4 Conclusions

Given the critical importance of determining NO_3_^–^ and NO_2_^–^ levels in water and the ongoing need for sensors that enable on-site monitoring without requiring sophisticated equipment or trained personnel, we have developed a smart hand-held nanosensor for easy and simultaneous environmental eMonitoring of NO_3_^–^ and NO_2_^–^ at the point of need. Our developed sensor integrates a paper-based nanosensing platform with a smart hand-held optical analyzer. The optical detection of NO_2_^–^ and NO_3_^–^ relies on fluorescence quenching of CQDs immobilized on the paper substrate within the sensor’s test zones in the presence of NO_2_^–^, along with the in situ reduction of NO_3_^–^ to NO_2_^–^ in the sensor’s reduction zone via a zinc-embedded paper channel. To enable smart quantification of analyte concentration detected by the paper sensor, a smart hand-held optical analyzer was also fabricated, equipped with a compact multispectral sensor/spectrometer for high-precision optical analysis, an IoT-enabled microcontroller for wireless connectivity and real-time data transfer, and a custom-developed smartphone app. The practical applicability of the developed sensor was validated by demonstrating its efficiency in the simultaneous monitoring of NO_2_^–^ and NO_3_^–^ across different water samples.

Our developed sensor offers several distinct advantages over previously reported NO_3_^–^ and NO_2_^–^ sensors, including: (I) ease of fabrication using ultralow-cost (≈$0.01 per sensor; Table S1) and nontoxic materials (paper, CQDs, and zinc), through equipment-free, green, low-cost, and reproducible methods—making it highly suitable for fabrication in resource-limited settings; (II) user-friendliness, with no special training required to perform the test; (III) enabling simultaneous detection of NO_3_^–^ and NO_2_^–^, facilitating identification of pollution sources and supporting targeted remediation strategies; and (IV) an affordable (≈$19.7; Table S1) hand-held optical analyzer featuring real-time wireless connectivity, enabling on-site and smart environmental monitoring of NO_3_^–^ and NO_2_^–^, even in remote or underserved areas.

Owing to its satisfactory performance and full alignment with the WHO’s REASSURED criteria for ideal sensing devices—outperforming many previously reported sensors for NO_3_^–^ and NO_2_^–^ detection (as shown in Table 2)—our developed sensor represents a promising solution for smart, simultaneous, and on-site environmental eMonitoring of NO_3_^–^ and NO_2_^–^ at the point of need, where are often far from centralized laboratory facilities.

**Table 2:**
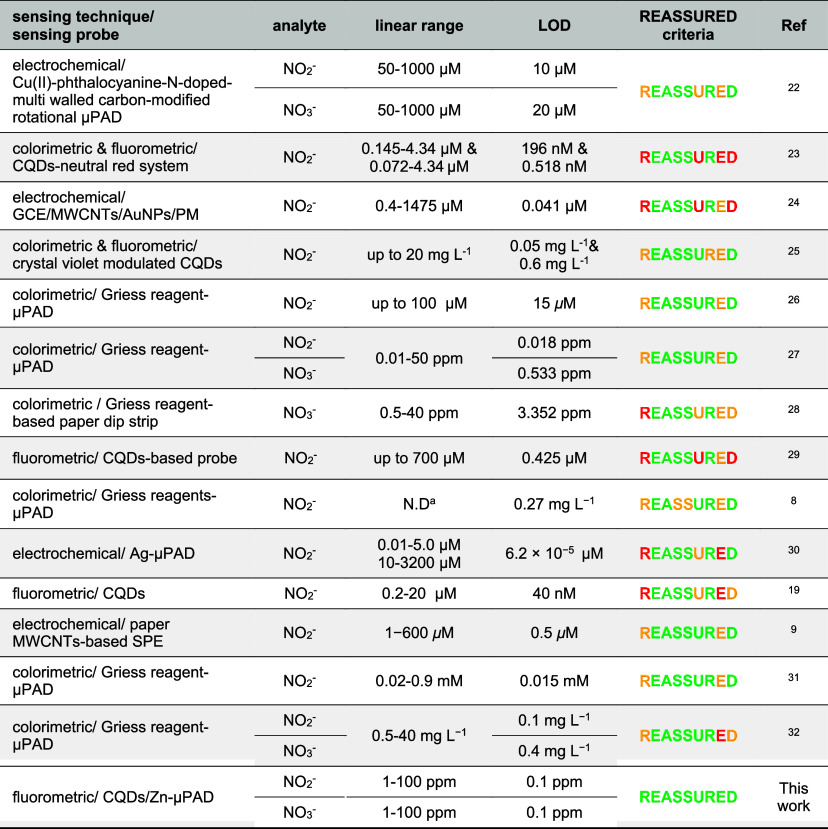
Comparison of Our Developed Sensor with Some of the Previously Reported NO_3_^–^ and NO_2_^–^ Sensors^22–32^ ^a^

a N.D.: not described. μPAD: microfluidic paper analytical device; CQDs: carbon quantum dots; GCE: glassy carbon electrode; MWCNTs: multiwalled carbon nanotubes; AuNPs: gold nanoparticles; PM: poly melamine; SPE: screen printed electrode. Color of REASSURED requirements/criteria indicates sensor opportunities in terms of REASSURED criteria: green, met; red, unmet; yellow, partially met.

## Supplementary Material



## Notes

TEM images, size histogram, and FTIR spectrum of the synthesized CQDs; effect of pH on the performance of the developed sensor; effect of zinc loading in the sensor’s reduction zone on NO_3_^–^-to-NO_2_^–^ conversion and performance of the developed sensor; response of the developed sensor over time; plot of fluorescence changes of the fabricated paper-based nanosensor over time; and estimated cost of the fabricated smart sensor, including the fabricated paper-based nanosensor and the fabricated smart hand-held optical analyzer (PDF)
