# Supplementary material for: Smart Paper-Based Nanosensor for Simultaneous Environmental eMonitoring of Nitrate and Nitrite
Source: ACS Meas Sci Au. 2025 Oct 30;5(6):972–80. doi: 10.1021/acsmeasuresciau.5c00122 (PMC12715739; doi:10.1021/acsmeasuresciau.5c00122)
Supplement: Supplementary file 1 [file tg5c00122_si_001.pdf]

Supporting Information

Smart Paper-based Nanosensor for Simultaneous Environmental eMonitoring of Nitrate and Nitrite

Mahdi Oroujlo[1,2], Zeinab Bagheri[1*], Tina Naghdi[2], Hamed Golmohammadi[2*]

[1] Department of Cell & Molecular Biology, Faculty of Life Sciences and Biotechnology, Shahid Beheshti University, 1983969411 Tehran, Iran

[2] Nanosensor Bioplatforms Laboratory, Chemistry and Chemical Engineering Research Center of Iran, 14335-186 Tehran, Iran

Corresponding authors email: ze_bagheri@sbu.ac.ir

golmohammadi@ccerci.ac.ir

Table of Contents

Figure S1: TEM images of the synthesized CQDs ………..…………………………………….S3

Figure S2: Size histogram of the synthesized CQDs……………...………………………....S4

Figure S3: FTIR spectrum of the synthesized CQDs..……………..………………….…….…S5

Figure S4: The effect of pH on the performance of the developed sensor in determination of 20 ppm NO_2^-……………………………………………..……………………………….………….. S6

Figure S5: The effect of zinc loading in the sensor's reduction zone on NO_3^--to-NO_2^- conversion and performance of the developed sensor in determination of 20 ppm NO_3^-…………………….S7

Figure S6: The response of the developed sensor over time in the presence of 20 ppm NO_2^-.S8

Figure S7: The plot of fluorescence changes of the fabricated paper-based nanosensor over time……………………………………………………...…………………………..…….….S9

Table S1: Estimated cost of the fabricated smart sensor, including the fabricated paper-based nanosensor and the fabricated smart handheld optical analyzer……...………S10

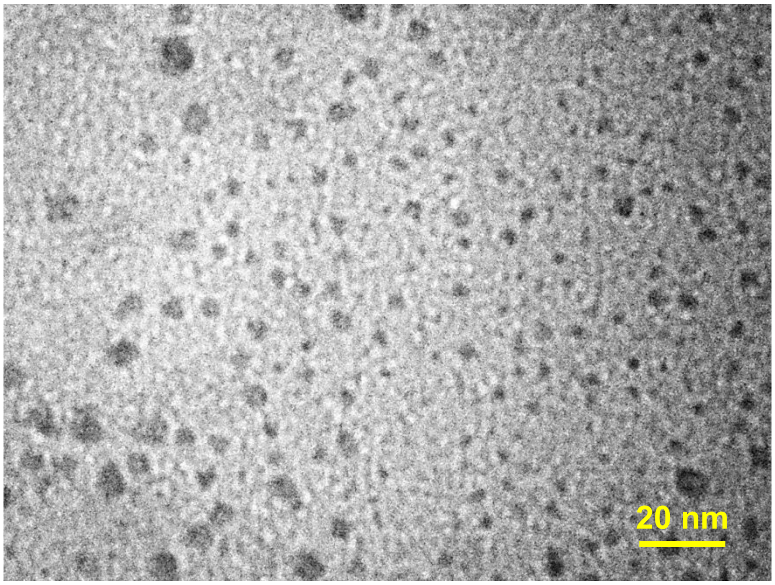

Figure S1: TEM image of the synthesized CQDs

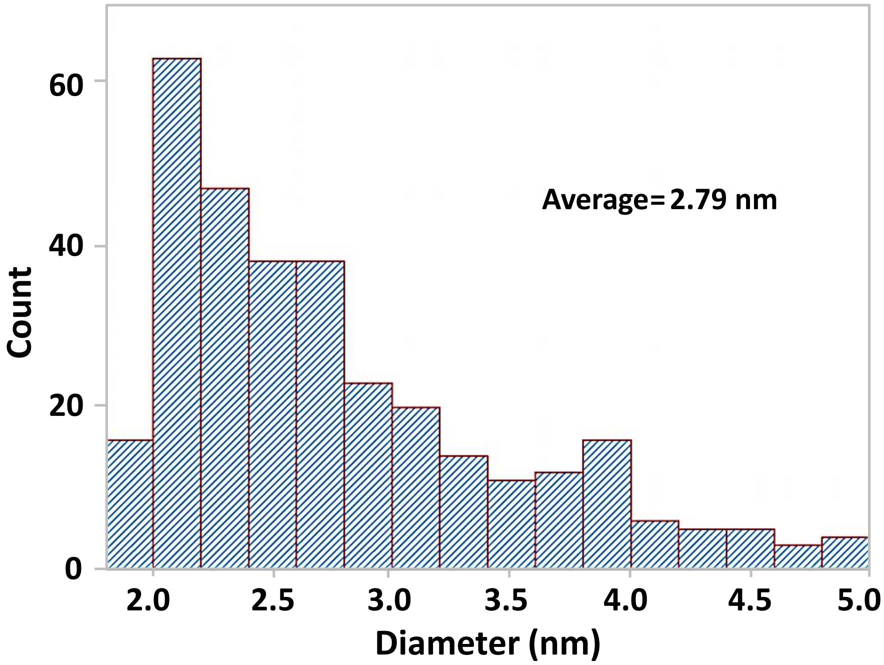

Figure S2: Size histogram of the synthesized CQDs

The TEM image and size histogram of the synthesized CQDs given in Figure S1 and S2 clearly reveal that they have been well dispersed with a spherical shape and the diameters in the range of 2–5 nm.

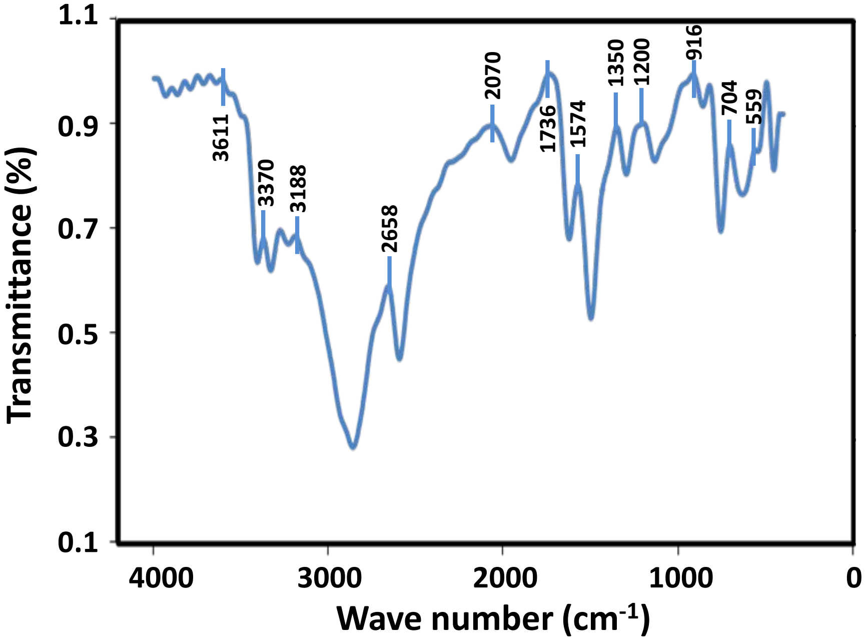

Figure S3: FTIR spectrum of the synthesized CQDs

The functional groups in the fabricated CQDs were identified by FT-IR analysis (Figure S3). The broad absorption bands around 3611, 370 and 3188 $cm^{-1}$are attributed to the stretching vibration of O—H/N—H groups, indicating the presence of surface –OH and/or $–NH_2$ functionalities. 2658 cm^{-1} band may be attributed to C–H stretching vibrations, possibly from aldehyde (–CHO), carboxylic acid groups (–COOH), or even residual hydrocarbon chains. The characteristic absorption peaks at approximately1736, 1574, and 1350 cm^{-1} are commonly assigned to amide I, II and III bands, respectively. The absorption peaks around 916 cm^{-1} are due to the C-H bending vibrations, especially from out-of-plane deformation in aromatic rings. The absorption bands ranging around 1200 cm^{-1} correspond to the asymmetric bridge oxygen and C—O stretching. These low-frequency bands around 704 and 559 cm-1 may correspond to C–H out-of-plane bending

or skeletal vibrations of substituted benzene rings or C–C bonds in a polyaromatic structure, which is correct considering that the CQDs are fabricated from O-phenylenediamine. As a result, the FT-IR spectrum confirms that the CQDs are rich in surface functional groups such as hydroxyl (–OH), carboxyl (–COOH), amine ($–NH_2$), carbonyl (C=O), and possibly aromatic domains. The good water solubility of fabricated CQDs can be due to the presence of various functional groups in their structure.

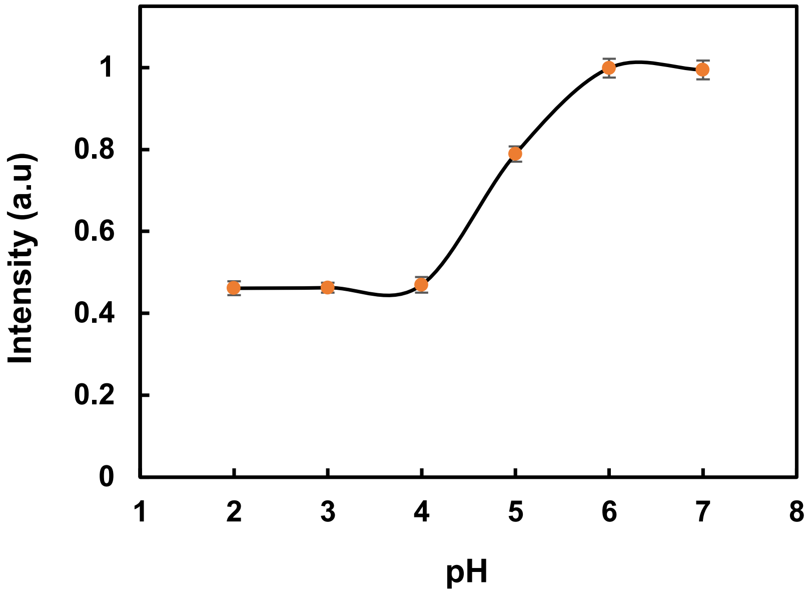

Figure S4: The effect of pH on the performance of the developed sensor in determination of 20 ppm NO_2^-. Error bars were obtained by taking the standard deviation of three tests (n = 3) in the same conditions.

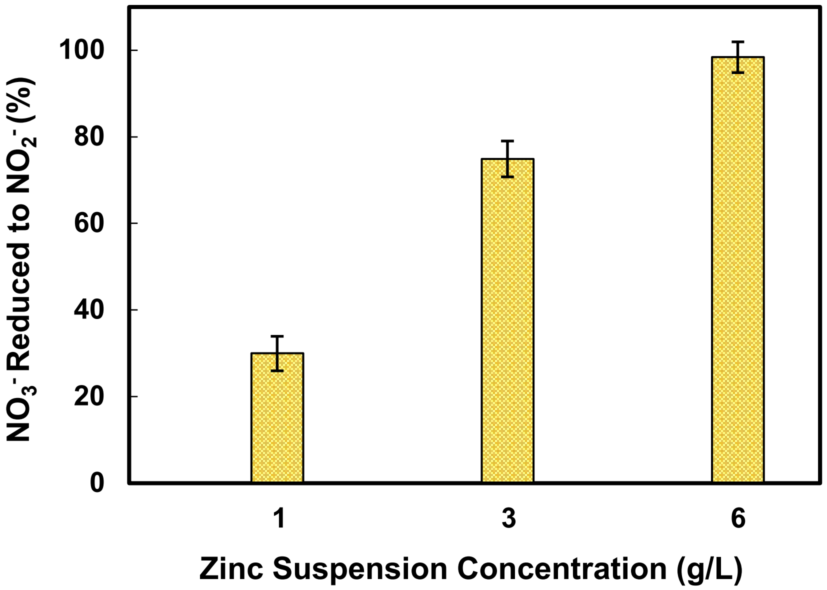

Figure S5: The effect of zinc loading in the sensor's reduction zone on NO_3^--to-NO_2^- conversion and performance of the developed sensor in determination of 20 ppm NO_3^-. Error bars were obtained by taking the standard deviation of three tests (n = 3) in the same conditions.

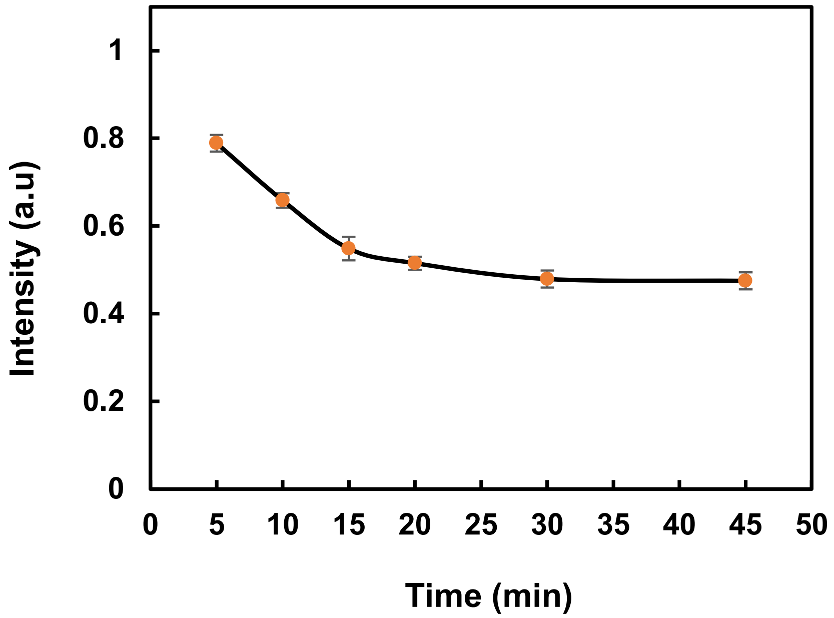

Figure S6: The response of the developed sensor over time in the presence of 20 ppm NO_2^-. Error bars were obtained by taking the standard deviation of three tests (n = 3) in the same conditions.

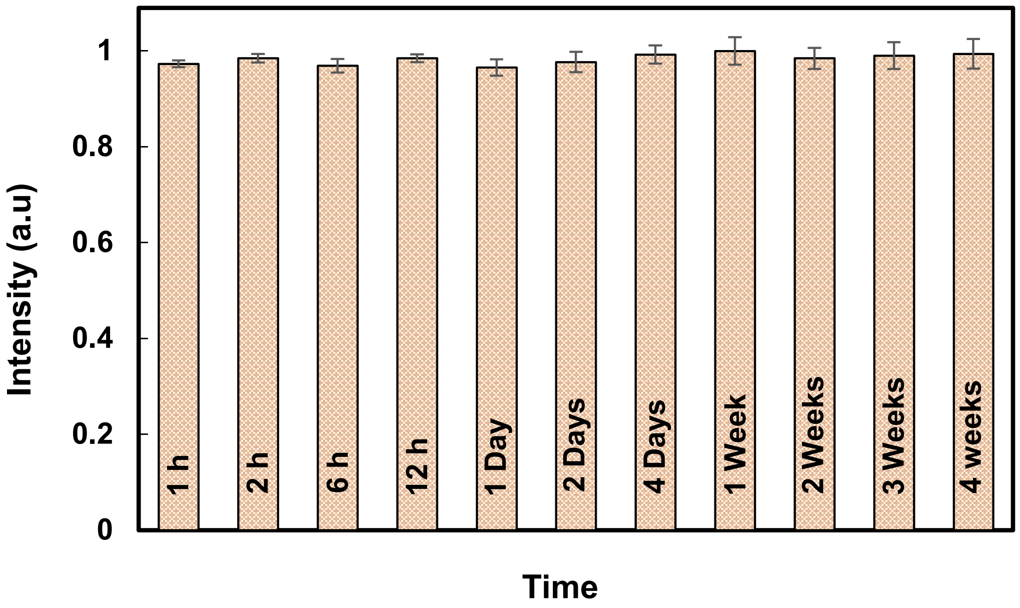

Figure S7: The plot of fluorescence changes of the fabricated paper-based nanosensor over time. Error bars were obtained by taking the standard deviation of three tests (n = 3) in the same conditions.

Table S1. Estimated cost of the fabricated smart sensor, including the fabricated paper-based nanosensor (above) and the fabricated smart handheld optical analyzer (below).

Material	Stock volume/amount	Price ($)	Amount required for each patch	Price for each patch ($)
O-phenylenediamine	500 g	75	1.5 mg	~ 0.001
Paper	Pack of 100	10.3	10 cm^2	~ 0.01
Zinc	250 g	66	0.6 mg	~ 0.001
				**Total ~ 0.01**

Smart handheld optical analyzer components	Price ($)
Multispectral sensor (Adafruit AS7341)	~ 11.7
ESP32 microcontroller module	~ 2
Rechargeable Li-ion battery	~ 1
Battery charging module	~ 1
OLED	~ 2
SMD UV-LED/emitter	~ 1.8
Plastic materials (ABS)	~ 0.2
	**Total ~19.7**
